# Preference-Based Determinants of Consumer Choice on the Polish Organic Food Market

**DOI:** 10.3390/ijerph191710895

**Published:** 2022-09-01

**Authors:** Agnieszka Dudziak, Anna Kocira

**Affiliations:** 1 Department of Power Engineering and Transportation, Faculty of Production Engineering, University of Life Sciences in Lublin, 20-612 Lublin, Poland; 2 Institute of Human Nutrition and Agriculture, The University College of Applied Sciences in Chełm, 22-100 Chełm, Poland

**Keywords:** sustainability, consumer attitudes, organic food, consumer behavior, sustainable food

## Abstract

**Background:** The development of the organic food market in Poland is currently at a fairly high level. There is a growing demand for organic food, but the share of total sales remains low. There are still many barriers related to the availability of organic food and information about it. In addition, consumers are skeptical of the inspection system in organic farming and admit that these foods do not meet their expectations regarding sensory qualities. **Methods:** The article conducted its own research, using an author’s survey questionnaire, which was distributed in Lublin Province. The research sample consisted of 342 respondents and was diverse in terms of gender, age and place of residence. The purpose of the analysis was to ascertain the determinants affecting the choice of organic food. For the study, the method of correspondence analysis was used, the purpose of which was to isolate characteristic groups of consumers who exhibit certain behaviors towards organic products. **Results:** Respondents admitted that they buy organic food several times a month, most often spending an amount of EUR 10–20 (per month). They also paid attention to product labeling, with labels read mostly by residents of small towns (up to 30,000 residents). Respondents were also asked about the reasons why they do not buy organic food. The results of the analysis show that respondents believe it is too expensive, but they also cannot point out differences with other products. **Conclusions:** The main purpose of this article was to study the preferences of organic food buyers and to identify factors that determine their choice but that may also be barriers to purchasing this category of food. These issues need to be further explored so as to create recommendations in this regard for various participants in the organic food market.

## 1. Introduction

Growing interest in various products offered on the organic food market is now observed, related not only to concerns about food safety [1] and sustainable agricultural production [2,3]. It is also motivated by the health impact of the diet [4]. This, in turn, encourages the introduction of healthy organic food into the food market, in line with the natural cycle [5,6]. In addition, the introduction of the Farm to Fork Strategy, a key component of the European Green Deal, whose overarching goal is to build a food chain that works for consumers, producers, the climate and the environment, is expected to enable the transition to a sustainable food system in EU countries while ensuring food security for people, as well as access to healthy food. This will ensure that Europeans have access to affordable and sustainable food with the support of measures to combat climate change, promote environmental protection, preserve biodiversity and support organic farming. One of the goals of the strategy is to allocate 25% of the EU’s arable land to organic farming, which, in addition to promoting sustainable food consumption and facilitating the transition to healthy, sustainable diets, can have a positive impact on the consumption of organic products [7].

Eyinade et al. [7] showed that consumer preferences for organic food are based on the general belief that it has more desirable characteristics than “traditional” produce [8].

However, the development of the market for organic products depends on the structure of sales channels, the level of prices, long-term trends in increasing standard of living and environmental awareness [9]. Furthermore, the diversified assortment caused by market development causes consumers to follow different opportunities related to purchasing or obtaining organic products, inducing competition in the organic market. In Poland, the organic food market is growing at 20% year to year and is one of the fastest growing sectors of the economy, and the growing competition balances its prices. Poland is perceived as an EU country with great potential for organic farming and the organic food market [10]. However, the products offered on the organic market should be approached with caution because, as the price of a product decreases, so does the quality. The growing demand is encouraging more and more specialized healthy food providers to emerge, and more and more Polish farmers are inclined toward ecology. This, in turn, causes new certified farms to emerge.

Consumer preferences for organic food are based on the general belief that it has more desirable characteristics than “traditional” produce. Consumer product choices are influenced by several factors, including status and lifestyle, as well as financial situation. Those who are not concerned with the origin of the produce can actually hinder the development of the organic market. On the other hand, consumers who are the most willing buyers of organic products treat the origin of products as part of a healthy lifestyle philosophy or a means to rational nutrition [11].

Furthermore, consumers are driven more by desires than by needs when purchasing food products. This forces them to search for additional features that add value to the product. For the organic food market, this means that meeting emotional needs is as important as ensuring the functional potential of the product. This requires creating an emotional bond with the consumer, who is more likely to purchase a product if they perceive it as a psychological and emotional benefit in addition to its expected functional properties. Therefore, the study of consumer preferences regarding organic food products should focus not only on the products’ attributes but also on the benefits they represent to the consumer [12].

This article aims to analyze the determinants that affect the choice of organic food by Poles to show the emerging trends in the Polish market related to the purchase of organic food.

## 2. Factors Determining Consumers’ Choice of Organic Market Products

A dynamic model of food quality, which assumes that a food product has functions determined by its properties, can help in understanding consumer perceptions of organic food. Properties are objective characteristics, independent of the user and determined by the composition of raw materials and conditions of the production process. Functions are subjective characteristics that relate to the product and exist in the interaction between the consumer and the product. They are also the main concern of the consumer, and, from the point of view of the manufacturer, it is important to control the properties and determine the relationship between functions and product properties. Appropriate shaping of product properties makes it possible to obtain the functional characteristics desired by consumers [13]. Product quality is multidimensional, and a product can be described by a universal set of attributes. In defining food product quality, four dimensions can be identified: hedonic, health, process and convenience. Hedonic quality refers to the pleasure of consumption based on sensory qualities, primarily taste, smell and appearance of the product. Health quality refers to the impact of the product on the consumer’s health. The quality dimension related to convenience consists of issues related to the processes of purchasing, storing, preparing and consuming the product. The process dimension is related to the characteristics of the production process and relates directly to the other quality dimensions [14].

There are many variations in the grouping of consumers with respect to food choice. One such example of consumer segmentation research was conducted by Roper Starch Worldwide, which approached the problem from the point of view of different consumer priorities. Their characteristics are shown in Table 1.

**Table 1:** Division of consumers by views on organic food.

Consumer Group	Group Characteristics
True Blues	They are politically active, want to have a say in current affairs and prefer not to use environmentally unfriendly products.
Greenback Greens	They have a strong value system but are not interested in political issues and also prefer to avoid environmentally unfriendly products.
Sprouts	For them, nature conservation is important but does not go hand in hand with their food choices.
Grousers	They are unwilling to change and know little about environmental protection. They consider organic products too expensive, yet they are not really different from conventional products.
Apathetics	They are completely uninterested in the natural environment, sustainability and “green” products.

[15,16,17,18,19,20,21,22].

The second division was created by the Natural Marketing Institute (NMI), who have also distinguished five different consumer groups. These characteristics are presented in Table 2.

Upon analyzing these two divisions, it can be observed that the results are very similar and the groups highlighted had similar priorities. The groups can be observed in all societies [16].

In the food market, many consumers are buying organic products, but there is also a large segment of the population that is not interested in buying them and does not identify with them for a number of reasons. One reason is the lack of clear differentiation between conventional and organic products and the higher price of the latter. According to a 2006 survey of Polish consumers, more than half of respondents who shopped for organic food were willing to pay only 10% more for organic food. Almost a fourth (23.5%) were willing to pay 11–25% more for healthy food and the rest of the respondents even more. It follows that price is a factor that is largely responsible for the level of sales of organic food as most consumers are not willing to spend more on it than on conventional food [23,24,25]. However, consumers often consider organic food to be a valuable alternative to popular conventionally produced food brands [26].

Organic food buyers are often advocates of regional food as a result of their views. They are in good shape and willing to take their time searching for the right food. They spend a large portion of their money on organic foods and are most likely to source them from specialized stores. In addition, they pay attention to the origin of the products and the ecological packaging [24]. According to some researchers, consumers pay attention to the origin of the product, and this information influences their purchasing decisions [27]. They divided the origin of food into “local” and “imported”, with the former perceived more positively. In contrast, Fehse et al. (2017) confirm that branding has a significant impact on the purchase decision of these products. Generally, branding is identified as environmental consciousness; in the eyes of customers, organic food is of higher quality [9,24]. Consumers relate the high quality of these products, also perceived as healthy foods, to their lifestyle habits, which has great potential as a foundation for marketing strategies [12].

Culture, or, more precisely, the principles learned in a family home, are of great importance among the factors that motivate and influence purchasing decisions. Poland is dominated by a traditional cuisine based on simple, unprocessed, all-available ingredients. However, the eating behaviors of Poles are shaped by economic and environmental factors that influence Polish culture and are related to consumption.

In Poland, the market for organic food is developing dynamically. Poland is also seen as a European Union country with a huge potential for organic farming, if only in terms of the area under organic farming and the number of organic farms. In addition, there is a growing number of conscious consumers in Poland convinced of its health benefits. Nowadays, more people are interested in product labeling, description and composition of product contents. Nevertheless, there is also a large group of consumers who, for many reasons, do not buy organic food, discouraged by, among other things, the high price or lack of confidence in products described as organic. Research conducted by Jarczok-Guzy indicates that the scope of promotion of organic food is narrow and prices are too high; moreover, organic food is difficult to access. Most consumers have heard of the food, but they mainly seek information on the Internet or by reading product labels, and there is still a large group of people who cannot correctly identify the labels for organic food [10].

Economic status has a huge impact on consumer choices and is related to education and social status. A relationship between income and food consumption was also observed. Often, people with a lower food budget pay attention to the price rather than the origin of food, while those with a higher budget pay attention to quality and origin, but also, although to a lesser extent, to price. Consumers with lower incomes often have to compromise on the quality and nutritional value of food, which contributes to a reduction in the pleasure derived from food shopping and consumption, linked to the awareness of one’s inability to choose from the ’better’ quality brands and products [28]. Marketing related to promoting healthy lifestyles is an important factor that influences consumer behavior. Consumers often look up information they have found on television, radio or online and thus become informed consumers. This situation changed significantly during the COVID -19 pandemic, when many food establishments were temporarily closed. Then, consumer spending on groceries increased and shifted largely to the Internet [29].

According to Shepherd et al. [30], in addition to the factors that influence the type of food purchased and its consumption, there are a variety of food-related considerations: aroma, texture, palatability and food buyer reasons. Equally important are the nutritional value of the product, its price, the broad range of available products and brand and product awareness among consumers [31].

Based on the observations of the organic food market, it can be concluded that Poles are motivated to buy organic products due to their beneficial health effects, taste and presentation of the product. Above all, they want to protect the environment that way [32]. However, it is not always environmental concerns that influence the purchase of these types of foods, as confirmed by Le-Anh and Nguyen-To [33]. On the other hand, Barrena et al. [12] found that the two main elements that determine the final choice to purchase organic foods are health and self-image. This is due to the health benefit effect, nutritional value and health safety guarantee of organic food, which are related to its perception as healthy food, ensuring healthy eating habits and quality of life, as well as security or peace of mind, dignity and self-respect. However, the reason why consumers choose conventional food over organic worldwide is because its price is too high, its availability is poor and it offers little variety [32].

Despite the abundance of information on consumer habits, it should be noted that it is difficult to clearly define the segment of the Polish consumer. Nevertheless, the profile is constantly changing. More and more people are aware of their food consumption needs. An interesting proposal for segmenting consumers in terms of the shopping habits and habits of Polish households was presented by Bilska et al. [34]. Nevertheless, the authors focused mainly on the problem of food waste. In the research conducted by Smiglak-Krajewska and Wojciechowska-Solis [35], the following groups of consumers were distinguished: eco-activists, eco-dietitians, eco-traditionalists and eco-innovators. An analysis of the motives for choosing organic products by selected types of consumers by Śmiglak-Krajewska and Wojciechowska-Solis shows that eco-activists pay more attention to marketing and practical features than to sensory attributes. Eco-dietitians, when deciding to buy organic products, take into account practical features first while paying slightly less attention to sensory features. A similar distribution of importance of the features of organic products can be noticed in the case of eco-traditionalists; however, the obtained values are lower. Sensory characteristics are of the least importance to the eco-innovator consumer type. A comparison of the groups of consumers showed that the most informed customers of organic products are eco-activists and eco-dietitians, who are able to notice all the benefits of organic products, which is reflected in the obtained values. Eco-activists and eco-innovators pay the most attention to marketing features and the least to sensory features, whereas eco-dietitians and eco-traditionalists pay the most attention to practical features and the least to marketing features [35].

## 3. Forecasted Directions of Changes in the Polish Organic Food Market

Since health-conscious communities are now emerging, organic producers are constantly looking for innovative solutions in agricultural production that could totally replace conventional ones. The growth of the organic food market is likely to involve the elimination of meat or the introduction of dairy substitutes. This is because consumers who prefer organic products also tend to have healthy eating habits that include many fruits and vegetables but less meat [36]. The Internet, especially social media, e.g., Instagram, Facebook, which educate the public, have a major impact on these changes, becoming the main source of information, entertainment and informal education and the chief communication space that shapes people’s tastes, knowledge and lifestyles [37]. Social media have been shown to play a major role in society, creating the meaning of diet and influencing food choices [38].

According to Adewuyi and Adefemi, together with the emergence of a green lifestyle, social media nowadays also become a crucial part of people’s daily life as they play an important role in spreading awareness of important information [39]. In their research, Nguyen and Zhang showed that social media influencers can moderate the intention–behavior gap within ecological lifestyle adoption by directly affecting consumers’ green behaviors. The influence includes the quality and quantity of contents, the authenticity and credibility from influencers and information and their personal background and characteristics [40]. The lack of trust and fear of conventional foods will increase the interest in the organic market, especially reliable and certified products.

Increasingly important in the organic market is the share of “free from food”, i.e., products in which specific ingredients were eliminated due to adverse effects on the human body (e.g., lactose-free, gluten-free or sugar-free). This is due to the increasing rate of diagnosed food intolerances and allergies. This trend is becoming more and more popular and is related to the wellness trend, which involves self-care. By 2021, its popularity was projected to grow at an average annual rate. However, consumers who do not have food allergies but care about the quality of the products they eat are also interested in “free from foods” [41].

Emerging technologies will allow every consumer to track a product “Farm to Fork” using a new generation of barcodes and blockchain. All it takes to find out the path a product has taken before it got into the consumer’s hands is a smartphone. According to public preference studies, consumers will prefer foods with a clear label and transparent packaging (if transparent packaging is used for the product). This is important for raw products (fruits and vegetables) for direct consumption because the freshness and quality of the product can be visually assessed, and this may influence the decision to purchase these foods by consumers who prefer healthy foods [2]. In 2018, an increased interest in a variety of supplements that have a positive impact on the health of the digestive system and mind can be observed [42,43]. The value of the market related to consumption and the proper functioning of the human body is bound to increase. Therefore, the willingness to purchase products that are beneficial to health will follow [28,29]. The popularity of various diets is also generating interest in edible insects, which contain high amounts of protein. Less surprising but increasingly popular are legumes. Vegans and vegetarians seek plant-based snack alternatives that resemble the taste of meat. Interest in such products has been growing since early 2019 [44,45]. This is why meat-based treat producers constantly expand the range and quantity of their offer [30,31].

The organic market is expected to grow steadily by 20% per year until 2030, which makes it a worthwhile investment. It can be expected that sales and availability of organic food will increase in the coming years [46,47,48,49,50].

In Central and Eastern Europe, a number of large-scale specialized organic food stores with a large selection of products and attractive prices are likely to appear. Moreover, small organic stores, offering only a selected category of products, e.g., only bread or vegetables, will become more and more popular. Moreover, online stores of large retail chains, such as Auchan or E. Leclerc, as well as Foodini.pl, have opened specialized “Eco” or “Bio” departments, offering organic food, and more and more brick-and-mortar organic stores are offering their products online. By innovating and creating online platforms, retailers communicate more effectively with consumers [51,52]. According to the study “Food Trade in Poland in 2010–2020” by Roland Berger, the food market will have to adapt to many factors related to the modern consumer. Food sales should combine traditional and online channels, taking into account products of the highest possible quality, grown in harmony with nature. This sales model fits best into the lifestyle of modern consumers who use technology to shop for groceries [53].

According to specialists, the organic market in Poland has positive prospects, and, in a few years, it should be on par with that in the EU. Consumers who want organic foods will not have a problem sourcing such products in Poland in the coming years.

## 4. Materials and Methods

A consumer study was conducted using a proprietary survey questionnaire (The Research questionnaire—Supplementary Materials). The research involved 342 respondents who were residents of Lublin Province in the south-eastern part of Poland. Grouping variables such as place of residence, age and gender were used to differentiate the respondents in a more detailed and additional way and were aimed at pointing to differences in the perception of the problem in the purchasing of organic products by consumers. The survey was conducted via the Internet, and the selection of respondents was random selection using the so-called the “snowball” method due to the fact that mainly young people use Internet resources; hence, their number turned out to be the largest.

The aim of the study was to analyze the determinants influencing the choice of organic food by the inhabitants of Lubelskie Voivodeship. However, since many respondents admitted that they buy food sporadically, in the further part of the analysis, the respondents were asked what the reasons for this situation were. Therefore, the analysis also includes the reasons for these negative attitudes towards organic food; the aim was to investigate the barriers that prevent consumers from buying organic food products.

The dominant group of respondents in this study were consumers aged 18–25, of which 68% were women (Table 3). Since consumers of organic products living in rural areas are less frequently analyzed in this type of research, this group of people constituted a large proportion of the respondents in the study (39%). The varied characteristics of the respondents allowed the demonstration of the differences in attitude toward the study subject and to show the relationship between the characteristics and their choices.

The survey was anonymous. The questions were related to the purchase of organic food products, the level of interest in them, reading the labels and knowledge of the packaging designation, the budget allocated for organic products and factors that encourage and discourage the purchase of this type of product and the attitude towards organic products. The responses allowed analyzing and evaluating the behavior of consumers in the organic food market and to interpret their attitude towards organic food products.

Data analyses were carried out on the basis of the statistical processing software Statistica 13.3 (Set Plus, version 5.0.96, license for University of Life Sciences in Lublin, Lublin, Poland) and Excel 2013 (Microsoft Office Professional Plus 2013, license for University of Life Sciences in Lublin, Lublin, Poland).

## 5. Results and Discussion

**Figure 1:**
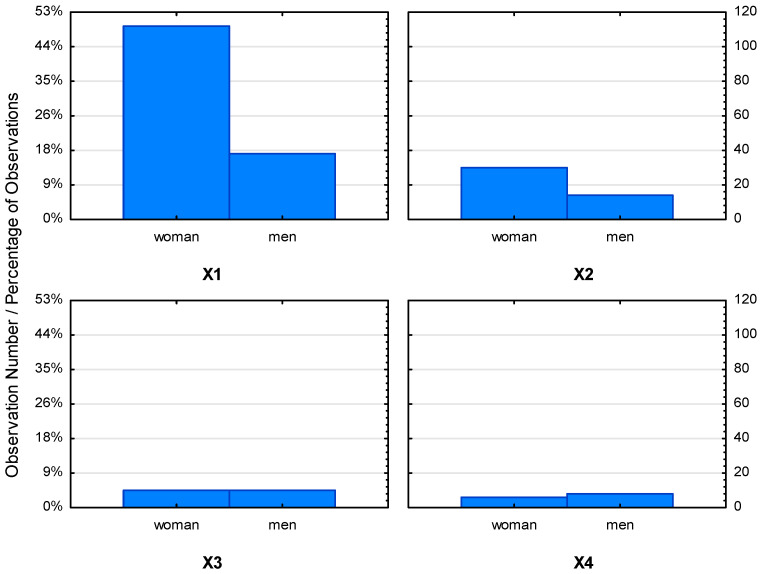
Graph of interaction of consumers declaring the purchase of organic food in relation to the sex of the respondents. (Abbreviations: X1—several times a month; X2—several times a year; X3—every day; X4—never).

Among the various issues raised in the research, the key issue was knowledge of organic products and the respondents’ declarations regarding their purchase. Most of the respondents declared that they knew and bought organic food. Figure 1 shows slight differences between individual groups of respondents depending on age and gender. However, among the largest group of respondents, i.e., aged 18–25, the majority of women declared that they buy organic food, while the opposite correlation was noted in men. Furthermore, there was greater interest in this type of product among women in two age groups, 26–40 years and 41–60 years old, who purchased only organic products. In the case of men, the age groups 18–25 and 41–60 purchased organic food more frequently. Please note that, in this study, women were twice as large a group of respondents as they are more often responsible for grocery shopping than men.

Table 4 provides more detailed information on the survey’s respondents by participation in each category of gender and place of living.

**Figure 2:**
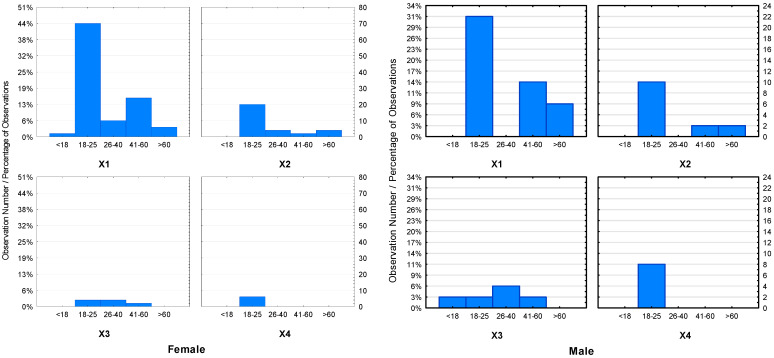
Graph of interaction of consumers declaring the purchase of organic food in relation to the sex and age of the respondents. (Abbreviations: X1—several times a month; X2—several times a year; X3—every day; X4—never).

The literature confirms that women buy organic food more often than men [54,55,56]. However, men have a higher level of awareness of organic food [57,58] and are more confident in their knowledge of organic products [59]. The results of our own research in relation to gender are shown in Figure 1. Taking into account the factor of age, it has been shown that younger consumers are more aware of organic food, prompting them to buy it more often [58,60]. For young people, a lifestyle based on organic food provides a sense of mental stability in life, a life lived in harmony with nature, history and their perception of health. Such a lifestyle ensures vitality as it relates its existence to the natural world [61]. Therefore, it is gaining more and more followers among young people. The results of our own research in relation to age and gender are shown in Figure 2.

The label on organic food packaging confirms that organic products are produced and processed according to the requirements related to the use of additives and artificial ingredients, pesticides, soil quality or the husbandry and processing of animal products. In addition, all ingredients and processing aids must be certified organic [62]. Given these stringent labeling requirements for organic products, it has been shown that most consumers have a broad and general understanding of what the name “organic product” on the packaging label means, including an understanding of how this food was produced and processed [62]. In terms of place of residence, it was found that consumers in cities with more than 30,000 residents prefer organic food, while consumers living in rural areas or cities with a population below 30,000 buy local rather than organic food [63,64,65].

**Figure 3:**
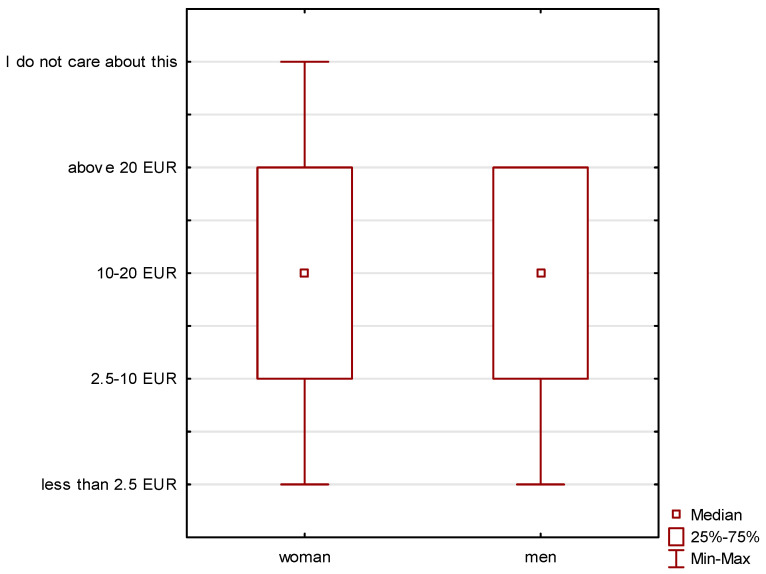
Declaration of the amount of money earmarked for the purchase of organic food depending on the gender of the respondents.

**Figure 4:**
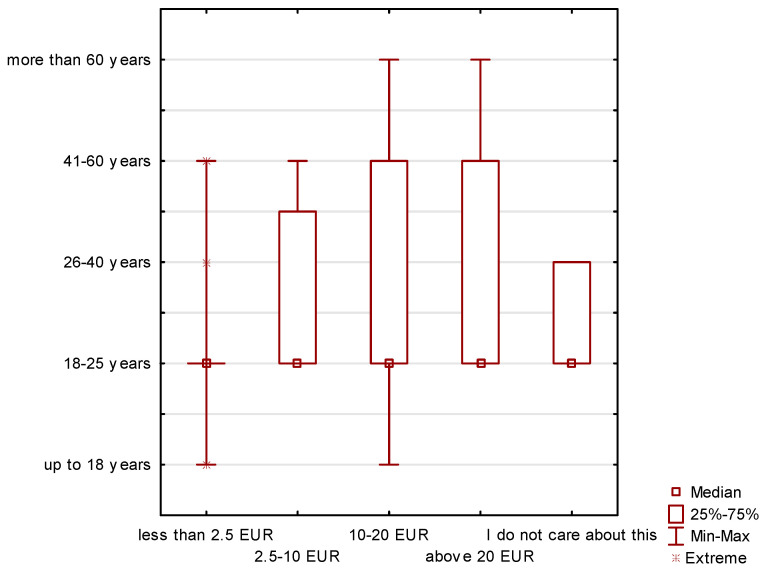
Declaration of the amount of money earmarked for the purchase of organic food depending on the age of the respondents.

The amount of funds allocated to the purchase of organic food is also an important issue. A clear differentiation can be observed in the group of respondents divided by gender (Figure 3), but, in division by age, these differences are small (Figure 4). Men declared specific amounts allocated to the purchase of organic food. On the other hand, some women declared that they were not interested in this issue.

Most often, the respondents spend EUR 10–20 (approximately PLN 45–90) of their monthly expenditure on organic food. Less than EUR 2.5 (approximately PLN 10) is spent mainly by people aged 18–25; this age group also often responded that they were not interested in spending money on organic products.

In the case of the declared expenditure of EUR 2.5–10 on organic products, three age groups, 18–25, 26–40 and 41–60, declared it. On the other hand, in the case of the amount exceeding EUR 20, it was declared in the age group of 18–60 years. Therefore, it is clearly visible that the budgets allocated to the purchase of organic food are not high.

### Statistical Analysis—Correspondence Analysis

Correspondence analysis (CA) is a multivariate statistical method for analyzing tables of categorial data or any data on a common ratio scale. Correspondence analysis is a descriptive and exploratory technique for analyzing two-way and multi-way tables containing certain measures that characterize the relationship between columns and rows. The obtained results provide information and allow for the analysis of the structure of qualitative variables making up the table. Therefore, as a result of the analyses, a two-dimensional contingency table was obtained, where the frequencies in the contingency table were first standardized in such a way that the relative frequencies were calculated, which, when summed up in all fields (cells) of the table, provide 1.0. One way to show the goals of a typical analysis is to express the relative frequencies by the distance between individual rows or columns in a space with a small number of dimensions. In correspondence analysis, inertia is defined as the quotient of the Pearson chi-squared statistic calculated from the two-way table by the total count (in the example presented, the total count is 342) [66].

Therefore, to analyze the market of organic products in Lubelskie Province, the correspondence between three groups of characteristics was analyzed: knowledge of the labels of organic products (three groups), place of residence (four groups) and gender (two groups). To present the configuration of the points representing the input data, a two-dimensional factor space was selected.

The first factor allows reproducing 80.04% of the input data variation (i.e., total inertia), and the second one 19.96% (Table 5).

The greatest share in a two-dimensional factor space was related to the knowledge of ecological product labels, namely the answers that respondents “sometimes” read labels and that they “did not” pay attention to it—coordinate I. On the other hand, in the case of coordinate II, the answers related to reading labels were: “always” and “sometimes”.

**Figure 5:**
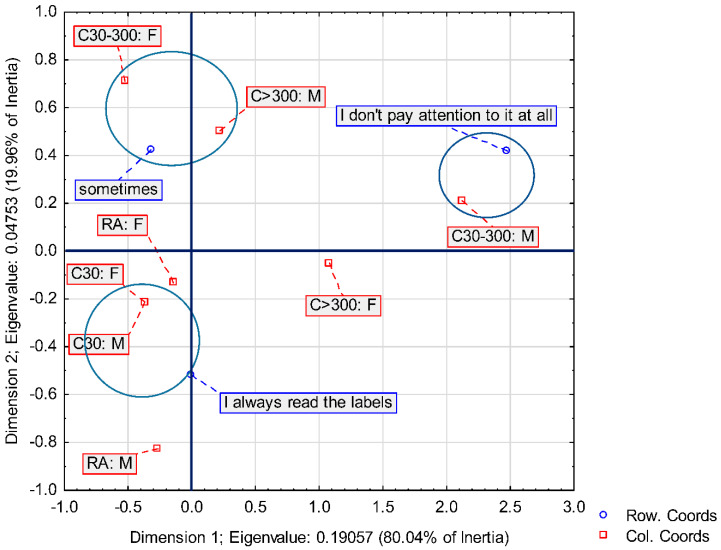
Correspondence analysis results between three groups of characteristics: knowledge of the labels on the packaging of organic products, place of residence of respondents and gender, canonical standardization. (Abbreviations: RA—rural area; C30—city to 30,000 residents; C30–300—city 30–300,000 residents; C > 300—city with more than 300,000 residents; Row. Coords—row coordinates; Col. Coords—column coordinates; F—female; M—male).

On the other hand, men living in a city of 30–300,000 and men living in a village had the largest share in the creation of a two-dimensional factor space, taking into consideration the place of residence and gender. Coordinate I was male residents of cities and coordinate II was male residents of rural areas (Figure 5).

The study distinguished three groups of consumers whose indicator structure depends on their interest in the label of the product they intend to buy (Figure 5). The first group (G1) is made up of people who “sometimes” read product labels and make a purchase. The second group (G2) includes customers who “do not” pay attention to the labels, and the third group (G3) is made up of people who “always” read the product labels. The fourth group consists of women living in rural areas and women living in large cities with more than 300,000 residents. This group’s structure is the closest to the average.

The strongest relationship was observed between people who “sometimes” read product labels: these are women living in cities of 30,000 to 300,000 residents and men living in cities with more than 300,000 residents. The group in question stands out from the others due to the index value of this factor. On the other hand, the respondents’ declaration that they “always” read labels is quite strongly related to men living in rural areas, as well as women and men living in small towns (up to 30,000 residents). In turn, consumers who “do not” pay attention to product labels are mainly men living in cities of 30–300,000 residents.

**Figure 6:**
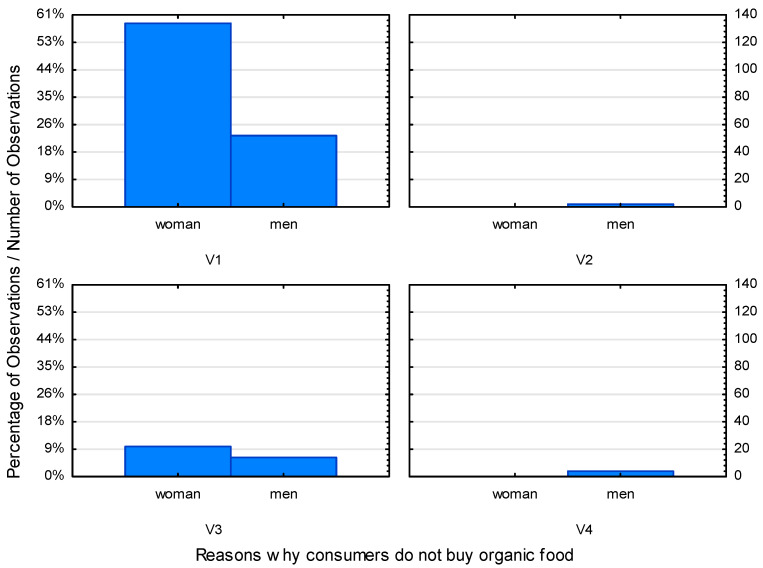
Categorized histogram describing the reasons that prevent consumers from buying organic food in relation to the gender of the respondents. (Abbreviations: V1—high price of eco food; V2—widespread availability of products from supermarkets; V3—no difference between products; V4—no interest in eco products).

As the research conducted earlier shows, purchases of organic products are still not a common phenomenon among Polish consumers. The main reasons for the lack of confidence of consumers in the rationality of purchasing organic food include, first of all, their high price. Other reasons include a lack of conviction about their nutritional value, or, as the respondents claim, “no difference” between organic and conventional products (Figure 6).

**Figure 7:**
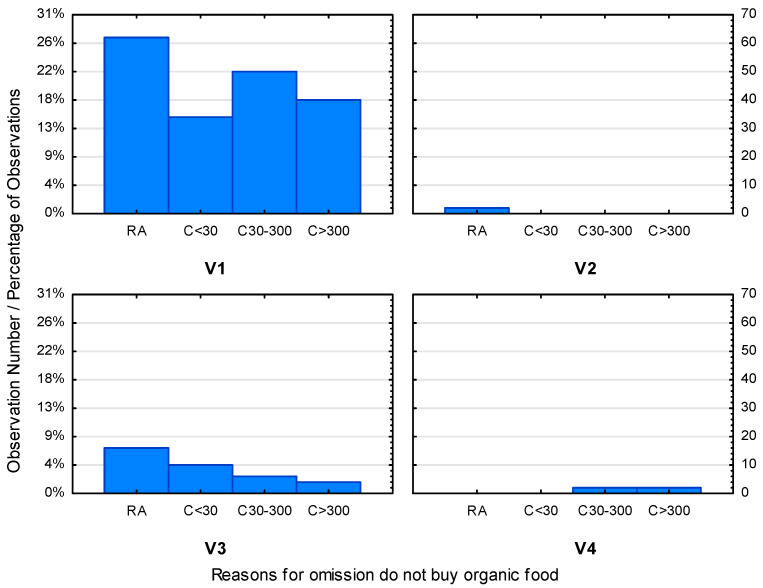
Categorized histogram describing reasons that prevent consumers from purchasing organic food in relation to where they live. (Abbreviations: V1—high price of eco food; V2—widespread availability of products from supermarkets; V3—no difference between products; V4—no interest in eco products; RA—rural area; C30—city to 30,000 residents; C30–300—city 30–300,000 residents; C > 300—city with more than 300,000 residents).

As shown in Figure 6 and Figure 7, the variables differentiating gender and place of residence clearly indicate that there is no differentiation in these two groups. Price remains the most significant factor regardless of the gender and place of residence of the respondents. Decisions to purchase organic products often depend on the budget allocated on such a purchase. Therefore, the lack of differences between an organic and a traditional product may not be a sufficient argument to buy the former.

Davies et al. [67] confirmed that the main factors that influence the purchase of organic products are their price and availability. In turn, Kyriakopoulos and Oude-Ophuis [68] argued that the quality of organic food determines its purchase to a greater extent than the price. Therefore, consumers’ knowledge and awareness of the benefits of organic food is important in the decision-making process. The inability to clearly distinguish between the two alternatives, as well as the price premium on organic produce, can complicate or influence the purchasing decision of the consumer in favor of cheap products [8,69].

However, the main factor influencing the choice of organic food is the consumer’s concern for their own health and that of their loved ones. Buyers have greater certainty as to the origin and the natural method of production of the produce when organic food is certified. Baer-Nawrocka and Szalaty (2017) concluded that the main motives behind consumers’ decision to purchase organic products are health considerations and the high quality of the products offered. This is demonstrated by numerous studies conducted among different groups of respondents indicating that health benefits are the most important rationale for purchasing organic food, which confirms that consumers are convinced of the health-promoting qualities of organic products [70,71,72,73]. On the other hand, concern for the environment as a reason for purchasing organic food was rated by consumers as an unimportant factor [74]. The implication is that motivation in choosing organic food is dominated by the perspective of individually perceived concern for health rather than concern for the natural environment [75].

The public is aware of the benefits of organic food, but this is often not reflected in the demand reported due to the higher price and lower availability of ecological products [59]. Organic food in specialized stores is purchased mainly by people with a higher monthly income, while people with secondary education buy organic food mainly at the bazaar due to the lower prices or the lack of specialized stores nearby. It follows that an additional barrier is hindered distribution, which is related to the fact that organic products usually have a short shelf life and, often, quick delivery to the consumer is a condition for their sale [74]. One of the barriers to the development of this market is the dispersion of supply [76]. Hence, the physical movement of products along the transportation and logistics chain plays a major role. In the market for organic products, short distribution channels that promote the sale of local products are often more advantageous from the point of view of producers because of the association with lower costs and margins, making it possible to sell products at competitive prices. At the same time, the risk of various types of damage or out-of-date food is reduced. As a result, direct sales, especially in the early stages of market development, based on sales in one’s own store, on an organic farm, at a market or agricultural retail trade are advantageous. It has been shown that the profitability of selling these products depends on the location of the organic farm near the main markets, which include large and very large urban areas. The strengths of direct sales are the control of the price level by the organic food producer, the adjustment of the offer to the structure and size of demand and the possibility of obtaining information on consumer expectations and preferences. However, this type of sale requires greater involvement on the part of the consumer and creates a greater sales risk burden for the producer [74,77].

## 6. Conclusions

The development of the organic food market can bring significant benefits not only to organic farmers, processors and intermediaries but also to customers and, ultimately, to society. The revival of organic farming and the organic food market should come from the actions of the governments of individual countries and the EU as a whole. In Poland, the organic food market is not yet as developed as in other EU countries, but, by learning from their practice and leveraging the profile and possibilities of organic farming, these differences can be minimized in the near future.

The most important conclusions resulting from the conducted research include:

- The majority of respondents declared that they know and buy organic food;
- Many respondents, however, admitted that they buy organic food occasionally;
- Among the largest group of respondents, i.e., those aged 18–25, most women declared that they buy organic food, while the opposite relationship was noted among men;
- There was greater interest in food among women in the two age groups of 26–40 and 41–60 who bought only organic products;
- For men, organic food was more frequently purchased by those aged 18–25 and 41–60 (women made up twice as large a group of respondents as they are more likely to be responsible for grocery shopping than men);
- With regard to place of residence, it was found that consumers in cities with more than 30,000 residents prefer organic food, while consumers living in rural areas or cities with less than 30,000 residents tend to buy local food rather than organic food;
- Most often, respondents spend EUR 10–20 of their monthly expenses on organic food. Less than EUR 2.5 is spent mainly by those aged 18–25 (this age group also often responded that they were not interested in spending money on organic products);
- For declared spending of EUR 2.5–10 on organic products, it was declared by the three age groups 18–25, 26–40 and 41–60, while, for the amount above EUR 20, it was declared by those aged 18–60. Thus, budgets for buying organic food are not high.

The practical implications of this study indicate that, as much as possible, action should be taken to convince consumers of the palatability, nutritional and health values of organic food so that the higher price comes second for the potential consumer. Highlighting the quality features of organic products will undoubtedly increase their demand and thus improve the health and general well-being of the population.

Please note that the tastes and preferences of consumers are changing, especially when it comes to the food market. Therefore, it is worth taking steps to produce food that meets the expectations of various consumer groups. Such recommendations should be particularly taken into account by producers and distributors. As the market is constantly developing, it must be researched both in terms of the health benefits of organic produce on the human body and the changing preferences of consumers. This is especially important in the age of comprehensive, all-available information as, these days, healthy eating has become a kind of fad. In light of the above, the growing awareness about organic products could contribute to the growth of the organic food market in Poland. Future research should focus on the level of consumer satisfaction, which affects the demand for organic products. Research into consumer personality types is also important, allowing the design and targeting of marketing strategies. The area of consumer expectations towards innovation in organic food is also worth investigating in the future.

## Figures and Tables

**Table 2:** Division of consumers according to the NMI.

Consumer Group	Group Characteristics
LOHAS (Lifestyles of Health and Sustainability)	They care about sustainability and healthy living. This group adopts an ecological lifestyle as its philosophy of life and wants to influence the environment with their actions.
Naturalites	They consider an active lifestyle and a healthy diet important to them, but this is not fully reflected in their purchasing decisions.
Conventionals	They are involved in environmental initiatives, but this is not their main concern. They choose food products that are attractively priced and will save them money.
Drifters	For them, environmental issues are a temporary fad. They want to be identified as environmentally conscious but do not apply the necessary principles in everyday life.
Unconcerneds	Similar to Apathetics, this group is not concerned with the environment and is not interested in organic foods, least in purchasing them.

[15,16,17,18,19,20,21,22].

**Table 3:** Socio-demographic profile of the respondents.

In Total	Number of Respondents	Percentage
	342	100.0
Gender:		
female	232	68.0
male	110	32.0
Age:		
up to 18 years	12	4.0
18–25 years old	236	69.0
26–40 years old	24	7.0
41–60 years old	46	13.0
60 years and more	24	7.0
Place of residence:		
rural area	132	39.0
city to 30,000 residents	48	14.0
30–300,000 residents	86	25.0
city with more than 300,000 residents	76	22.0

**Table 4:** Respondents’ attitudes toward organic food by gender and place of living.

		Gender		Place of Living			
		F *n* (%)	M *n* (%)	RA *n* (%)	C30 *n* (%)	C30–300 *n* (%)	C300 *n* (%)
Buying Organic Food	yes	150 (61.2)	58 (27.8)	76 (36.5)	44 (21.2)	50 (24.0)	38 (18.3)
	no	82 (61.2)	52 (38.8)	56 (41.7)	4 (3.0)	36 (26.9)	38 (28.4)
Frequency of Organic Food Purchases	every day	10 (50.0)	10 (50.0)	10 (50.0)	4 (20.0)	2 (10.0)	4 (20.0)
	several times a month	112 (74.7)	38 (25.3)	50 (33.3)	34 (22.7)	38 (25.3)	28 (18.7)
	several times a year	30 (68.2)	14 (31.8)	18 (40.9)	6 (13.7)	10 (22.7)	10 (22.7)
	never	6 (42.9)	8 (57.1)	2 (14.2)	2 (14.3)	4 (28.6)	6 (42.9)
Amount of Money Spent on Organic Food Purchases (Per Month)	<EUR 2.5	6 (100.0)	0 (0.0)	0 (0.0)	2 (3.3)	4 (66.7)	0 (0.0)
	EUR 2.5-10	50 (67.6)	24 (32.4)	26 (35.2)	16 (21.6)	10 (13.5)	22 (29.7)
	EUR 10-20	56 (70.0)	24 (30.0)	26 (32.5)	20 (25.0)	24 (30.0)	10 (12.5)
	>EUR 20	12 (50.0)	12 (50.0)	14 (58.4)	0 (0.0)	2 (8.3)	8 (33.3)
	I do not care about this	30 (78.9)	8 (21.1)	14 (36.8)	6 (15.8)	12 (31.6)	6 (15.8)
Reasons to Stop Buying Organic Food	too expensive	134 (72.0)	52 (28.0)	62 (33.3)	34 (18.3)	50 (26.9)	40 (21.5)
	are no different from any other food	22 (61.0)	14 (39.0)	16 (44.4)	10 (27.8)	6 (16.7)	4 (11.1)
	widespread availability of products from supermarkets	0 (0.0)	2 (100.0)	2 (100.0)	0 (0.0)	0 (0.0)	0 (0.0)
	no interest in organic food	0 (0.0)	4 (100.0)	0 (0.0)	0 (0.0)	2 (50.0)	2 (50.0)
Reading Labels of Organic Products	yes	66 (66.0)	34 (34.0)	44 (44.0)	22 (22.0)	16 (16.0)	18 (18.0)
	sometimes	80 (74.1)	28 (25.9)	36 (33.3)	22 (20.4)	32 (29.6)	18 (16.7)
	I don’t pay attention at all	8 (57.1)	6 (42.9)	2 (14.3)	0 (0.0)	4 (28.6)	8 (57.1)

Abbreviations: F—female; M—male; RA—rural area; C30—city up to 30,000 residents; C30–300—city 30–300,000 residents; C300—city of more than 300,000 residents.

**Table 5:** Information resources factors.

Number of Dimensions	Eigenvalues and inertia, Total inertia = 0.23810 χ^2^ = 52.859 df = 14 *p* = 0.0000				
	Singular Value	Eigenvalues	Percentage of Inertia	Cumulative Percentage	χ^2^
1	0.436548	0.190574	80.03881	80.0388	42.30747
2	0.218009	0.047528	19.96119	100.0000	10.55123

## Data Availability

Not applicable.

## Supplementary Materials

The following supporting information can be downloaded at: https://www.mdpi.com/article/10.3390/ijerph191710895/s1, Research Questionnaire.
