# Supplementary material for: Preference-Based Determinants of Consumer Choice on the Polish Organic Food Market
Source: Int J Environ Res Public Health. 2022 Sep 1;19(17):10895. doi: 10.3390/ijerph191710895 (PMC9518508; doi:10.3390/ijerph191710895)
Supplement: Supplementary file 1 [file ijerph-19-10895-s001.zip › ijerph-1862051-supplementary.pdf]

Supplementary Materials

Research Questionnaire

1) Do you buy organic food?
   a) yes
   b) no
2) How often do you buy organic food?
   a) every day
   b) several times a month
   c) several times a year
   d) never
3) How would you rate your knowledge of organic food?
   a) good
   b) hard to say
   c) not interested in it
4) What do you think is the motive for buying organic products?
   a) buying them is fashionable
   b) they have a good effect on health
   c) they have a good brand name
   d) they taste better than regular products

5. How much money do you spend on organic food in a week?
   a) less than 2.5-10 EUR
   b) from 2.5-10 EUR
   c) from 10-20 EUR
   d) more than 20 EUR
   e) I do not care about this
6. Do you use additional information about organic food?
   a) yes
   b) no

7) Where do you gather information on organic food from?
   a) Internet
   b) friends
   c) press/books
   d) nutritionist
8) What do you think are the characteristics of organic food?
   a) it is expensive
   b) it is healthy
   c) is high quality
   d) is tasty
   e) is not tasty

9. How do you assess the availability of organic food on the Polish market?
   a) poorly available
   b) easily available
   c) unavailable
10. Which factor do you think could increase consumption of organic products?

a) lower price
b) better availability
c) increased prevalence
d) more attractive packaging

11. What do you think is the reason that keeps you from buying organic food?
a) the price of food is too high
b) no difference between organic and conventional food
c) I am not interested in organic food
d) widespread availability of products from supermarkets

12. When buying organic food, do you pay attention to the signs and information on packaging?
a) yes
b) sometimes
c) I don't pay attention at all

METRICS

1. Gender
a) woman
b) man

2. Age
a) up to 18 years
b) 18-25 years
c) 26-40 years
d) 41-60 years
e) more than 60 years

3. Place of residence
a) rural area
b) city up to 30 th. residents
c) city of 30-300 th. residents
c) city of more than 300 th. residents
